# Associations of snack frequency, energy density and nutritional quality with diet quality and cardiometabolic risks in adolescents: National Health and Nutrition Examination Survey 2009–2016

**DOI:** 10.1017/S0007114525105746

**Published:** 2026-01-14

**Authors:** Binyam Girma Sisay, Kathleen E. Lacy, Sarah A. McNaughton, Rebecca M. Leech

**Affiliations:** 1 Institute for Physical Activity and Nutrition, School of Exercise and Nutrition Sciences, Deakin University, Melbourne, VIC, Australia; 2 School of Exercise and Nutrition Sciences, Deakin University, Melbourne, VIC, Australia; 3 Health and Well-Being Centre for Research Innovation, School of Human Movement and Nutrition Sciences, University of Queensland, St Lucia, QLD 4067, Australia

**Keywords:** Adolescents, Snack, Diet quality, Adiposity, Metabolic syndrome risk

## Abstract

To examine the association between snack characteristics (snack frequency, snack energy density and snack nutritional quality) with diet quality and cardiometabolic risks among US adolescents from the 2009–2016 National Health and Nutrition Examination Survey. Cross-sectional dietary data collected using a 24-h dietary recall from the 2011–2016 National Health and Nutrition Examination Survey (1999 boys and 1897 girls aged 12–19 years) were analysed. Associations between snack characteristics with diet quality, fasting blood glucose, TAG, total cholesterol, HDL, LDL, blood pressure, waist circumference and metabolic syndrome risk score using multiple linear regression were analysed stratified by sex. Higher snack nutritional quality (*β* (95 % CI): boys 0·31 (0·09, 0·52); girls 0·44 (0·30, 0·57)) was linked to better overall diet quality, whereas snack energy density excluding beverages (*β* (95 % CI): boys –1·82 (–2·52, –1·12); girls –1·75 (–2·69, –0·82)) was linked to poorer overall diet quality. Among girls, higher snack frequency was associated with lower waist circumference and lower fasting blood glucose (–0·67 (–1·28, −0·05)). Additionally, higher snack energy density and nutritional quality were associated with lower waist circumference and TAG, respectively. No associations between snack characteristics and cardiometabolic indicators or metabolic syndrome risk score were observed for boys. Findings suggest that strategies to improve adolescent snack nutritional quality and energy density may enhance overall diet quality. However, limited associations were observed between snack characteristics and cardiometabolic risk indicators among girls only. Prospective studies are needed to further investigate the relationship between snack characteristics and adolescent health outcomes.

Cardiometabolic risk (CMR) refers to a clinical abnormality that predicts chronic diseases, such as cardiovascular diseases and type 2 diabetes. CMR includes abdominal obesity, elevated blood glucose, elevated blood pressure (BP), raised TAG and reduced HDL-cholesterol^(1)^. In recent years, a growing body of evidence has identified CMR among adolescents and young adults^(2)^. In 2020, an estimated 35·5 million adolescents worldwide, representing 4·8 % of the adolescent population, were living with metabolic syndrome^(3)^. Metabolic syndrome is a cluster of interrelated individual factors including elevated adiposity, fasting blood glucose, TAG, BP and low HDL that collectively increase the risk of cardiovascular diseases and type 2 diabetes^(1)^. Among US adolescents, the prevalence of elevated waist circumference (WC) and blood glucose has increased significantly from 1999 to 2018, rising from 12·47 to 17·59 % and from 10·07 to 23·65 %, respectively^(2)^. Notably, elevated BP and blood glucose levels in this age group have been linked to an increased risk of premature mortality in adulthood^(4)^. Existing reviews have consistently shown that unhealthy dietary patterns, including increased intake of sodium, saturated fat, meat, fast food and sugar-sweetened soft drinks, have been significantly associated with elevated CMR^(5)^.

Snacking is common among adolescents (10–19 years), contributing up to one-quarter of their overall energy intake (EI)^(6)^. Adolescents’ food choices at snacks typically consist of energy-dense and nutrient-poor foods, with fruits and dairy products contributing a smaller proportion^(7)^. Snacks are significant sources of added sugar, saturated fat and sodium, potentially increasing CMR in this population^(8)^. On the other hand, selecting nutrient-dense choices such as fruits and dairy foods as snacks may help regulate appetite and improve cardiometabolic health^(9)^. Meanwhile studies in adults have demonstrated that regular consumption of almonds at snacks can lower LDL^(10)^, blood glucose and EI^(9)^. Conversely, consuming snacks of poor nutritional quality is associated with increased fasting TAG, postprandial TAG, fasting insulin and insulin resistance in adults^(11)^. Snack nutritional quality remains an under-studied area owing to the lack of established tools to specifically examine the nutritional quality of snacks.

A cross-sectional study of British adolescents by Murakami and Livingstone^(12)^ found that eating frequency was positively associated with BMI z-scores, but no associations were observed for CMR. However, this study did not differentiate between meal and snack frequency to investigate the relationships with CMR. Furthermore, there is little research exploring the association between snack characteristics such as frequency, energy density (ED) and snack nutritional quality, assessed using a comprehensive scoring method, and CMR in adolescents. Analysing these snack characteristics might yield insight into the role of snacks in adolescent cardiometabolic health. Such understanding could inform the development of targeted messages and interventions aimed at promoting healthier snacking and improving metabolic health among adolescents. Therefore, this study aims to examine the association between snack characteristics (snack frequency, snack ED and snack nutritional quality) with diet quality and CMR among US adolescents from the 2009–2016 National Health and Nutrition Examination Survey (NHANES).

## Methods

### Sample and study design

This study employed a cross-sectional design that incorporated data from the 2009–2010, 2011–2012, 2013–2014 and 2015–2016 NHANES cycles. The NHANES was a nationally representative survey of the US population that included civilians not residing in institutionalised settings. The survey excluded individuals under institutional supervision or custody, active-duty military personnel, their family members living overseas and US citizens residing outside the fifty states and the District of Columbia. Participants were selected using a complex, multistage probability sampling design with the county as the primary sampling unit, from which clusters of participants were randomly chosen^(13)^. Written parental consent was obtained from all adolescents aged 12–17 years, and assent was obtained from the adolescents themselves. Written informed consent was obtained from adolescents aged 18–19 years. The NHANES study protocol was approved by the National Center for Health Statistics Research Ethics Review Board. The data has been accessed for the purpose of this study on 01 May 2024. This study received ethics exemption (Registration ID: 2023-184) from the Deakin University Human Research Ethics Committee on 22 June 2023. The present study utilised the first 24-h dietary recall day, anthropometric measurements (WC, height and weight), BP measurements and biomarkers (fasting blood glucose, HDL, LDL, TAG and total cholesterol).

### Dietary assessment

NHANES dietary data were obtained using the Automated Multiple-Pass 24-h dietary recall method developed by the US Department of Agriculture^(14)^. Initial dietary intake information was collected through in-person interviews for the first 24-h period, followed by a second recall collected via telephone within 3–10 d after the initial recall. Participants were asked to specify the time of day they consumed each food and beverage and to classify each eating occasion (EO) using a predetermined list of categories. Self-reported dietary data were obtained from adolescents aged 12–19 years old without the assistance or presence of an adult. Data collection primarily involved the use of the English language during interviews. However, in cases where the study respondent spoke only Spanish or faced difficulty comprehending the questions or concepts, a Spanish-speaking interviewer conducted the interview. In situations where the respondent spoke a language other than English, such as Chinese, French or Japanese, or lacked sufficient proficiency in English to conduct the interview, a household interpreter, friend or neighbour was enlisted to assist. The interpreter was required to be at least 18 years of age^(15)^.

### Snack definition

In the present study, EO reported by participants as ‘snacks’, ‘drinks’ or their Spanish equivalents were classified as snacks. Conversely, EO in which the participant reported as ‘breakfast’, ‘brunch’, ‘lunch’, ‘supper’, ‘dinner’ or their Spanish equivalents were classified as meals. EO that were reported by participants as ‘extended consumption’ or ‘other/don’t know’ were categorised as either meals or snacks if they occurred concurrently or within a 15-min window of the EO that the participant identified as a meal or snack. EO with an EI of less than 210 kJ were excluded, and any EO that occurred within 15 min of each other were combined and treated as a single EO^(16)^. This approach, which is based on the participant’s identification of EO and the additional criteria of a 15-min time interval and a minimum EI of 210 kJ, has been shown to be the most effective in predicting variations in total EI among children and adolescents^(16)^.

### Characteristics of snacks

The snack characteristics examined in this study were snack frequency, snack ED and snack nutritional quality. The snack frequency per day was estimated using the participant-identified snack definition (as described above). Two different methods were used to determine the ED of snacks. The first method included all foods and beverages, whereas the second method excluded liquids consumed as beverages. The calculation of ED, excluding beverages, followed the method proposed by Vernarelli *et al.*^(17)^. Specifically, beverages containing energy and those without energy were excluded, but tap water and milk (including non-dairy milk substitutes) were treated differently because they are often consumed as ingredients or as additional components in other food items (e.g. milk added to ready-to-eat cereals or tap water added to cooked cereals). Milk and water consumed as ingredients were included as foods in the ED calculations. The daily total energy and weight derived from snacks were first determined to calculate the ED (including all foods and beverages and excluding beverages). Next, the total energy from the snack was divided by the corresponding total weight to obtain the ED.

Snack nutritional quality was assessed using the hybrid nutrient density score (HNDS), as described in a previous publication^(18)^. Briefly, the HNDS score was calculated for each food and beverage consumed, considering three aspects: ‘nutrients to encourage’, ‘food groups to encourage’ and ‘nutrients to limit’. The ‘nutrients to encourage’ include protein, fibre, vitamin D, potassium, Ca and Fe. The ‘food groups to encourage’ encompass whole grains, vegetables, fruit, dairy products and nuts and seeds. In contrast, the ‘nutrients to limit’ include sodium, total sugars and saturated fats. The HNDS was calculated by combining the relative contribution to the daily value of beneficial nutrients (capped at 100 %) and the sum of the percentage contributions of recommended food groups based on a 2000-calorie diet and then subtracting the sum of the percentage contributions of nutrients and components to limit, relative to their daily value. The HNDS of snacks ranges from 97 (most healthy) to 29 (least healthy), with higher scores indicating better nutritional quality.

To calculate the HNDS of foods and beverages for snacks, the HNDS of each food and beverage was multiplied by its energy content. These values were then summed for each snack or meal, and the resulting total was divided by the total EI of snacks. This method was based on the procedure outlined by Murakami^(19)^.

Food groups consumed at snacks were classified using the What We Eat In America Food Groups^(20)^ and Food Pattern Equivalent Database^(21)^ classification (online Supplementary File 2). The percentage contribution of snacks to daily intakes of solid fats, added sugars, oils, refined grains, protein foods, dairy products, fruits, whole grains and vegetables was also calculated.

### Diet quality

The overall quality of adolescent diet was assessed using the Healthy Eating Index (HEI-2015), which evaluates the alignment of an individual’s dietary choices with the Dietary Guidelines for Americans 2015–2020^(22)^. The HEI-2015 consists of thirteen components, with nine assessing the sufficiency of dietary intake: total fruits, whole fruits, total vegetables, greens and beans, whole grains, dairy products, total protein foods, seafood and plant proteins, fatty acids and refined grains. The remaining four components evaluate dietary moderation, including refined grain, sodium, added sugar and saturated fats. The individual components are summed to produce the HEI-2015 score, with a maximum value of 100. A higher score indicates better diet quality^(23)^.

### Evaluation of energy intake reporting

Potential energy misreporting was assessed using a method developed by Huang *et al.*^(24)^, which involves calculating the ratio of EI to estimated energy requirement (EER). EER was calculated using the established dietary reference intake equation, considering sex, age, body weight, height and physical activity level^(25)^. However, due to the lack of accurate physical activity data for adolescents, we assumed a ‘low-active’ level (≥ 1·4 to < 1·6) for all participants. This assumption is supported by national data indicating that only 24 % of children aged 6–17 years and 26·1 % of high school students engage in 60 min of physical activity daily in the USA^(26)^. Assuming low physical activity levels is likely to have a minimal impact on estimated EER values. The EI:EER ratio was included as a covariate in the regression models to adjust for energy misreporting.

### Anthropometric, cardiometabolic measures and cardiometabolic risk score

Trained technicians measured height, weight and WC in a mobile examination centre following a standard protocol^(27)^. WC was measured using a measuring tape at the uppermost lateral border of the hip crest (ilium) to the nearest 0·1 cm^(27)^.

Systolic and diastolic BP were measured after adolescents had been seated quietly in a resting position for 5 min. Three consecutive BP readings were taken on the same hand. If the examiner was unable to hear one or more of the readings (systolic or diastolic), a fourth attempt may be made. Mean BP measurements were calculated and used in the present analysis^(27)^.

During the mobile examination centre visit, blood samples were collected and analysed for fasting blood glucose, TAG, LDL, HDL and total cholesterol in the subsample of adolescents who attended the morning session and had fasted for at least 9 h.

The metabolic syndrome risk score was calculated using the equation developed by Gurka *et al.*^(28)^, which takes into account sex, race/ethnicity, BMI z-score, systolic BP, fasting blood glucose, TAG and HDL.

### Covariates

This study examined several covariates, including age, racial/ethnic group, meal nutritional quality, average weekly metabolic equivalent (MET), HEI-2015, BMI z-score and survey cycle. Meal nutritional quality was assessed using the HNDS, following the same procedures as for snack nutritional quality. In addition, meal ED was assessed using the method described above for snack ED. Adolescent age was reported in complete years. The Centers for Disease Control and Prevention’s (CDC) 2000 BMI for age growth charts were used to calculate adolescents’ age- and sex-specific BMI z-score^(29)^. Self-identified racial/ethnic groups were categorised as follows: (a) Mexican American, (b) non-Hispanic White, (c) Other Hispanic and (d) non-Hispanic black. The survey cycle was also included as a covariate, with the following periods: (a) 2009–2010, (b) 2011–2012, (c) 2013–2014 and (d) 2015–2016 to account for changes in snack characteristics, cardiometabolic factors and diet quality throughout the years.

In the 2009–2016 NHANES, physical activity was assessed using the Global Physical Activity Questionnaire^(15)^, assessing the frequency and duration of physical activity of participants in three domains: (1) work-related activity, (2) leisure-time physical activity and (3) leisure-time physical activity^(30)^. In accordance with previous recommendations, vigorous-intensity activities, comprising vigorous work-related and leisure-related activities, were classified as 8·0 MET. Moderate-intensity activities, including moderate leisure-related activities and active transportation (walking or bicycling), were assigned a value of 4.0 MET. To estimate each participant’s total physical activity, the number of days, average time and corresponding MET were multiplied and then summed^(31)^. We used the average weekly total MET-weighted minutes spent engaging in moderate-to-vigorous physical activities over a typical week as a measure of individual physical activity level.

### Analytical sample

The present study included adolescents aged 12–19 years who had completed their initial 24-h dietary recall, ensuring both a maximised sample size and the maintenance of national representativeness. Initially, 4908 adolescents were eligible for inclusion in this study. However, 1012 adolescents did not report consuming a snack on their first day’s 24-h dietary recall and were excluded. The final analytical sample comprised 1999 boys and 1897 girls. Additionally, adolescents with missing values for BP, fasting blood glucose, TAG, HDL, WC or BMI were excluded from the models using one of these CMR indicators as a dependent variable. A schematic representation of the final sample determination is provided in online Supplementary File 1.

### Data analysis

All statistical analyses were performed using the appropriate NHANES person and replicate weights to account for the probability of selection and complex sampling design^(32)^. Descriptive statistics were performed using proportions with corresponding 95 % CI for categorical variables and means with corresponding 95 % CI for continuous variables. The mean difference was assessed using an F test, whereas differences in proportions were tested using the adjusted Wald test.

Multiple linear regression models, stratified by sex, were used to examine the association between diet quality (HEI-2015), metabolic syndrome risk score, fasting blood glucose, TAG, total cholesterol, HDL, LDL, systolic BP, diastolic BP, WC, with snack characteristics (snack ED, snack frequency and snack nutritional quality). All models were adjusted for age, socio-economic position (Poverty-Income Ratio (PIR)), average weekly MET, EI:EER, survey cycle, BMI z-score (except for the model including WC), HEI-2015 (all models except when HEI-2015 is an outcome or snack nutritional quality is an independent variable) and meal frequency. For models where snack ED was the independent variable, these were also adjusted for the respective meal ED. For models with snack nutritional quality as the independent variable, these were also adjusted for meal nutritional quality. All model assumptions have been checked. Statistical significance was set at *P* < 0·05.

To understand the representativeness of the present study’s analytic sample of adolescent snack consumers, we did a sensitivity analysis examining the differences in sociodemographic characteristics, CMR indicators and CMR scores between snack consumers and non-consumers. Additionally, we investigated the associations between snack frequency, diet quality, CMR indicators and CMR score, including participants with zero snack consumption (total sample 4908 adolescents; online Supplementary File 3), to explore the impact of excluding those with no snack consumption for our analysis.

## Results

Table 1 presents the characteristics of adolescents in the NHANES 2009–2016. The results showed no significant differences in mean age, snack nutritional quality and ED of snacks (with and without beverages) between boys and girls. However, boys had significantly higher mean WC, metabolic syndrome risk score, fasting blood glucose, TAG, systolic and diastolic BP compared with girls. In contrast, girls had higher HEI-2015, HDL and LDL compared with boys. In addition, there were no significant differences in sociodemographic characteristics or metabolic syndrome risk scores between snack consumers and non-snack consumers. However, snack consumers had higher TAG levels and HEI-2015 scores, while non-snack consumers had higher WC and BMI z-scores (online Supplementary File 3).

**Table 1.** Characteristics of adolescents (12–19 years) in the National Health and Nutrition Examination Survey 2009–2016

Characteristics	Boys (*n* 1999)		Girls (*n* 1897)		P^*^
	Mean	95 % CI	Mean	95 % CI	
Age (years)	15·4	15·2, 15·5	15·4	15·2, 15·5	0·99
Race and ethnicity (%)					
Mexican American	14·0	11·4, 17·0	14·0	11·4, 17·0	0·55
Other Hispanic	6·5	5·1, 8·1	7·5	6·0, 9·2	< 0·001
Non-Hispanic White	57·4	52·4, 62·4	55·9	51·1, 60·5	< 0·001
Non-Hispanic Black	13·7	11·1, 16·9	14·2	11·5, 17·5	< 0·001
Other Race – Including Multi-Racial	8·5	7·1, 10·1	8·0	6·5, 9·8	< 0·001
Ratio of family income to poverty (%)					
< 125 %	27·9	24·0, 32·2	30·4	26·4, 34·8	0·13
≥ 125 %	72·1	67·8, 76·0	69·6	65·2, 73·6	< 0·001
Fasting blood glucose (mg/dl)	96·7	94·9, 98·5	92·2	91·4, 93·1	< 0·001
TAG (mg/dl)	84·7	79·1, 90·2	77·0	72·2, 81·8	0·0321
HDL (mg/dl)	49·7	48·8, 50·6	53·7	52·8, 54·6	< 0·001
LDL (mg/dl)	86·2	83·5, 88·9	89·6	87·8, 91·3	0·03
Total cholesterol (mg/dl)	153·4	158·9, 162·4	153·4	151·6, 155·3	< 0·001
Systolic blood pressure (mmHg)	111·3	110·7, 111·9	105·9	105·3, 106·5	< 0·001
Diastolic blood pressure (mmHg)	60·1	59·1, 61·1	58·9	57·9, 59·9	< 0·01
Waist circumference (cm)	82·1	81·1, 83·1	81·1	80·2, 82	0·12
BMI z-score	0·5	0·4, 0·6	0·6	0·5, 0·7	0·24
Metabolic syndrome risk score	3·1	3·0, 3·1	2·4	2·3, 2·5	< 0·001
Snack frequency (*n*/d)	2·1	2·0, 2·1	1·9	1·9, 2·0	0·01
Energy density (ED)					
ED of snacks including beverages (kcal/g)	0·2	0·2, 0·3	0·2	0·2, 0·3	0·7
ED of snacks without beverages(kcal/g)	0·4	0·4, 0·5	0·5	0·4, 0·5	0·68
Snack nutritional quality	2·1	1·8, 2·5	2·4	2·0, 2·8	0·23
HEI-2015	45·1	44·4, 45·8	46·3	45·6, 47·0	< 0·01

HEI, Healthy Eating Index.

* *P*-value for differences between boys and girls based on F test (continuous variable) or *χ*^2^ test (categorical variables).

Snacks account for nearly one-quarter of saturated fats and sodium and more than one-quarter of added sugars. Conversely, over one-third of daily fruit intake comes from snacks. Among food groups consumed at snack, the highest mean intakes include both nutritious and less nutritious choices: sugar-sweetened beverages, total milk, fruits and other desserts (online Supplementary File 2).

Table 2 presents the associations between snack characteristics and diet quality among adolescents in the NHANES 2009–2016. Higher nutritional quality of snacks, as assessed by the HNDS, was associated with higher overall diet quality, measured by the HEI-2015, in both boys and girls. Specifically, a one-point increase in the HNDS was associated with a 0·31-point and 0·44-point increase in HEI-2015 scores in boys and girls, respectively. Conversely, snacks with higher ED (except for ED including beverages in girls) were associated with lower diet quality in both sexes.

**Table 2.** The association between snack characteristics and diet quality among adolescents (12–19 years) in the National Health and Nutrition Examination Survey 2009–2016

	Boys		Girls	
	*β*	95 % CI	*β*	95 % CI
Snack frequency (*n*/d)^†^	0·57	–0·02, 1·16	0·88	0·16, 1·6
Energy density (ED)^‡^				
ED of snacks including beverages (kcal/g)	−1·91^**^	**−3·02, −0·80**	−0·21	−1·25, 0·83
ED of snacks without beverages (kcal/g)	−1·82^***^	**−2·52, −1·12**	−1·75^***^	**−2·69, −0·82**
Snack nutritional quality^§||^	0·31^**^	**0·09, 0·52**	0·44^***^	**0·3, 0·57**

*P* values: *P < 0·05, **P < 0·01, ***P < 0·001.

† These results, obtained from multiple linear regression, were further adjusted for age, race and ethnicity, family income to poverty ratio, survey cycle, meal frequency and ratio of energy intake to estimated energy requirement (EI:EER).

‡ These results, obtained from multiple linear regression, were further adjusted for age, race and ethnicity, family income to poverty ratio, survey cycle, snack frequency, meal frequency, respective meal energy density and EI:EER.

§ These results, obtained from multiple linear regression, were further adjusted for age, race and ethnicity, family income to poverty ratio, survey cycle, snack frequency, meal frequency, meal nutritional quality and EI:EER.

|| Snack nutritional quality assessed by hybrid nutrient density score. The score ranges from −15 (least healthy) to 89 (most healthy).

Tables 3 and 4 present the association between snack characteristics, cardiometabolic indicators and metabolic syndrome risk score of adolescent boys and girls, respectively, in the NHANES 2009–2016. Among boys, there was no association between snack characteristics and cardiometabolic indicators and metabolic syndrome risk score (Table 3). In contrast, among girls, higher snack frequency and ED were associated with lower WC, and higher snack nutritional quality was associated with lower TAG. Furthermore, higher snack frequency was associated with lower fasting blood glucose among girls only (Table 4). Sensitivity analysis of the associations between snack frequency, diet quality, CMR and CMR scores after including those with no snack consumption revealed findings consistent with those of our primary analysis (which excluded non-snackers), except for a lack of association between snack frequency and fasting blood glucose among girls in the sensitivity analysis (online Supplementary File 3).

**Table 3.** The association between snack characteristics, cardiometabolic indicators and metabolic syndrome risk score among adolescent boys (12–19 years) in the National Health and Nutrition Examination Survey 2009–2016

	Fasting blood glucose (mg/dl)		TAG (mg/dl)		Total cholesterol (mg/dl)		HDL (mg/dl)		LDL (mg/dl)		Systolic blood pressure (mmHg)		Diastolic blood pressure (mmHg)		Waist circumference (cm)		Metabolic syndrome risk score	
	*β*	95 % CI	*β*	95 % CI	*β*	95 % CI	*β*	95 % CI	*β*	95 % CI	*β*	95 % CI	*β*	95 % CI	*β*	95 % CI	*β*	95 % CI
Snack frequency (*n*/d)^*^	−0·72	−2·42, 0·99	0·01	−0·03, 0·05	0·01	0·00, 0·02	0·09	−0·58, 0·75	0·05	−2·89, 2·99	−0·29	−0·88, 0·30	−0·48	−0·95, 0·00	0·00	−0·01, 0·01	0·02	−0·02, 0·06
Energy density (ED)^†^																		
ED of snacks including beverages (kcal/g)	−0·02	−1·02, 0·98	−0·02	−0·08, 0·05	0·00	−0·02, 0·03	0·92	−0·02, 1·86	2·78	−4·77, 10·33	−0·13	−1·5, 1·25	−0·41	−1·87, 1·04	0·00	−0·02, 0·02	0·01	−0·05, 0·07
ED of snacks without beverages (kcal/g)	0·96	−0·5, 2·42	0·02	−0·03, 0·07	0·00	−0·01, 0·02	0·74	−0·4, 1·88	3·10	−1·29, 7·48	0·03	−0·65, 0·71	0·01	−0·82, 0·84	0·00	−0·01, 0·01	0·03	−0·02, 0·08
Snack nutritional quality^‡§^	−0·03	−0·08, 0·02	0·00	0·00, 0·00	0·00	0·00, 0·00	0·04	−0·05, 0·13	0·16	0·00, 0·32	−0·04	−0·1, 0·01	0·02	−0·03, 0·06	0·00	0·00, 0·00	0·00	0·00, 0·00

Waist circumference (WC) and TAG were log-transformed to improve normality. The format for interpretation of the b-coefficient estimates is therefore 100 × (coefficient), corresponding to the percentage change for a 1-unit increase in the independent variable (while holding all other variables constant).

* These results, obtained from multiple linear regression, were further adjusted for age, race and ethnicity, family income to poverty ratio, survey cycle, meal frequency, BMI z-score (except in model including WC as an outcome), average weekly metabolic equivalent and ratio of energy intake to estimated energy requirement (EI:EER).

† These results, obtained from multiple linear regression, were further adjusted for age, race and ethnicity, family income to poverty ratio, survey cycle, snack frequency, meal frequency, respective meal energy density, Healthy Eating Index -2015, BMI z-score (except in model including WC as an outcome), average weekly metabolic equivalent and EI:EER.

‡ These results, obtained from multiple linear regression, were further adjusted for age, race and ethnicity, family income to poverty ratio, survey cycle, snack frequency, meal frequency, meal nutritional quality, BMI z-score (except in model including WC as an outcome), average weekly metabolic equivalent and EI:EER.

§ Snack nutritional quality assessed by hybrid nutrient density score. The score ranges from −14 (least healthy) to 86 (most healthy).

**Table 4.** The association between snack characteristics, cardiometabolic indicators and metabolic syndrome risk score among adolescent girls (12–19 years) in the National Health and Nutrition Examination Survey 2009–2016

	Fasting blood glucose (mg/dl)		TAG (mg/dl)		Total cholesterol (mg/dl)		HDL (mg/dl)		LDL (mg/dl)		Systolic blood pressure (mmHg)		Diastolic blood pressure (mmHg)		Waist circumference (cm)		Metabolic syndrome risk score	
	*β*	95 % CI	*β*	95 % CI	*β*	95 % CI	*β*	95 % CI	*β*	95 % CI	*β*	95 % CI	*β*	95 % CI	*β*	95 % CI	*β*	95 % CI
Snack frequency (*n*/d)^†^	−0·67^*^	**−1·28, −0·05**	−0·01	−0·05, 0·04	0·01	0·00, 0·02	0·73	0·00, 1·46	1·65	−0·08, 3·38	−0·30	−0·88, 0·28	−0·09	−0·69, 0·51	−0·02^**^	**−0·03, −0·01**	0·00	−0·04, 0·04
Energy density (ED)^‡^																		
ED of snacks including beverages (kcal/g)	0·11	−0·73, 0·95	0·02	−0·04, 0·09	−0·01	−0·03, 0·01	0·69	−0·41, 1·79	1·03	−1·5, 3·56	0·03	−0·65, 0·71	−0·22	−1·2, 0·76	−0·02^*^	**−0·03, 0·00**	0·04	−0·02, 0·11
ED of snacks without beverages(kcal/g)	−0·01	−0·78, 0·75	−0·01	−0·06, 0·04	−0·01	−0·02, 0·00	0·76	−0·06, 1·59	−1·49	−3·83, 0·85	0·13	−0·45, 0·71	−0·20	−0·84, 0·45	−0·02^***^	**−0·03, −0·01**	0·00	−0·06, 0·06
Snack nutritional quality^§||^	−0·07	−0·14, 0·01	−0·01^**^	**−0·02, 0·00**	0·00	0·00, 0·00	0·15	−0·03, 0·33	−0·33	−0·73, 0·08	−0·02	−0·1, 0·06	−0·02	−0·12, 0·07	0·00	0·00, 0·00	0·00	−0·01, 0·01

Waist circumference (WC) and TAG were log-transformed to improve normality. The format for interpretation of the b-coefficient estimates is therefore 100 × (coefficient), corresponding to the percentage change for a 1-unit increase in the independent variable (while holding all other variables constant).

*P* values: *P < 0·05, **P < 0·01, ***P < 0·001.

† These results, obtained from multiple linear regression, were further adjusted for age, race and ethnicity, family income to poverty ratio, survey cycle, meal frequency, BMI z-score (except in model including WC as an outcome), average weekly metabolic equivalent and ratio of energy intake to estimated energy requirement (EI:EER).

‡ These results, obtained from multiple linear regression, were further adjusted for age, race and ethnicity, family income to poverty ratio, survey cycle, snack frequency, meal frequency, respective meal energy density, Healthy Eating Index-2015, BMI z-score (except in model including WC as an outcome), average weekly metabolic equivalent and EI:EER.

§ These results, obtained from multiple linear regression, were further adjusted for age, race and ethnicity, family income to poverty ratio, survey cycle, snack frequency, meal frequency, meal nutritional quality, BMI z-score (except in model including WC as an outcome), average weekly metabolic equivalent and EI:EER.

|| Snack nutritional quality assessed by hybrid nutrient density score. The score ranges from −15 (least healthy) to 89 (most healthy).

## Discussion

To our knowledge, this is one of the first studies to examine the relationship between snack characteristics and CMR in adolescents. Among boys and girls, lower snack ED and higher snack nutritional quality were associated with better diet quality. Among girls only, higher snack frequency was associated with lower WC and lower fasting blood glucose. In contrast, no associations were found for snack characteristics with CMR indicators among boys. The observed sex differences in the association between snack characteristics and CMR may be attributed to differences in energy requirements, pubertal timing and physical activity and energy expenditure^(33)^. Overall, the findings from this study suggest that promoting consumption of lower ED snacks with higher nutritional quality may improve overall diet quality among adolescents.

Snack nutritional quality, assessed using comprehensive nutrient profiling systems such as the Nutrient Profiling Scoring Criterion^(34)^, British Food Standards Agency nutrient profiling system^(19)^, snack diet index^(11)^ and nutrient density score, is emerging as an important characteristic of snacks. Our findings showed that higher snack nutritional quality, evaluated using the HNDS, was associated with better diet quality. This finding is consistent with our previous study among Australian adolescents, which used the Nutrient Profiling Scoring Criterion to assess snack nutritional quality^(34)^. The observed association may be attributed to overlapping components between the HEI-2015 and the HNDS, including intake of vegetables, whole grains, nuts and seeds, fruit, sodium, sugar and saturated fats^(18)^. Indeed, when snacks were removed in the calculation of the HEI-2015, the association between snack nutritional quality and diet quality was attenuated, further supporting the contribution of snacks to overall diet quality (data not shown). Snacks account for nearly one-quarter of total saturated fat, added sugar and sodium intake, while also providing over one-quarter of daily fruit consumption, thereby contributing both positively and negatively to overall diet quality. On the contrary, there was no association between snack nutritional quality and cardiometabolic indicators or CMR score among boys and girls except for a weak association with TAG among adolescent girls. The association between individual nutrients and CMR factors, such as central adiposity and CMR score, has been shown to vary by age and sex. Consequently, nutrient profile scores should also account for these differences^(35)^. However, the currently used nutrient profiling scores are not age- and sex-specific, which may have contributed to the observed lack of association. Additionally, the nutrient density score used to assess the comprehensive nutritional quality of snacks was not specifically designed to evaluate the nutritional composition of snacks in relation to optimal cardiometabolic health in adolescents.

Snack ED is another characteristic of snacks that is related to adiposity^(36)^ and the tendency for lower ED diets to be associated with higher consumption of vegetables, fruits and dietary fibre^(37)^. Among US adults, dietary ED was also associated with elevated insulin resistance^(38)^. Our findings indicate that higher snack ED, excluding beverages, was associated with lower diet quality in both boys and girls. Additionally, among boys, snack ED including beverages was also associated with diet quality. These findings align with research by Murakami and Livingstone^(6)^, which demonstrated that higher ED snacks (identified using time-of-day and energy contribution approaches) were linked to lower diet quality and reduced consumption of vegetables, fruits and dietary fibre among British adolescents. We also found that snack ED among girls had a weak association with lower WC and Waist-to-Height Ratio (WHtR). In contrast, Murakami and Livingstone^(6)^ reported no association between snack ED, BMI z-score and WHtR in their study. Our present study adjusted for overall diet quality, whereas the study by Murakami and Livingstone^(6)^ did not, which might explain the difference in our findings^(6)^. Arango-Angarita *et al.*^(36)^ revealed inconsistent findings regarding the association between dietary ED and overweight/obesity indices, likely due to variations in dietary ED calculation methods. In the present study, we used two ED calculation methods – one including beverages and one excluding beverages – making our findings comparable to previous studies and allowing us to explore the role of different ED calculations on the association with CMR among adolescents.

Snack frequency has been the most studied characteristic of snacks in relation to diet quality and adiposity. However, examining snack frequency alone may provide only limited insights into the nutritional quality or composition of snacks. Studies have consistently shown that higher snack frequencies are associated with increased EI, which likely leads to elevated adiposity and adverse health outcomes^(39)^. Our findings indicate that higher snack frequency was not associated with better diet quality or lower WC. Previous studies have reported inconsistent findings regarding the relationship between snack nutritional quality and diet quality in adolescents^(40)^. This inconsistency may be due to the use of varying methods to define snacks. In the present study, a participant-identified approach, coupled with a 15-min time interval and a 210-kJ minimum, was used to identify snacks, as this method has previously been shown to predict the most variation in total EI among children and adolescents^(16)^. Additionally, we found that in girls, higher snack frequency was linked to lower fasting blood glucose levels. This association may be attributed to the regulatory effect of consumption of commonly consumed nutritious foods in snacks (e.g. dairy foods) on blood glucose, as supported by previous studies^(41)^. The HNDS assigns equal weight to all food groups to encourage (whole grain, vegetables, fruit, dairy products and nuts and seeds) in calculating the final score. However, this approach may overlook the varying impacts that different food groups have on adolescent cardiometabolic health. In contrast, the HEI allocates different scores to each food group, aiming to better reflect each food group’s unique contribution to overall health outcomes and the prevalence of dietary inadequacies within the population. However, aside from the association described above, no other significant associations were observed. This may be due to the limited informativeness of snack frequency regarding the nutritional content of snacks. Snack frequency may have limited utility in assessing the relationship between snacks and cardiometabolic indicators.

This study possesses several notable strengths. First, it comprehensively explored the characteristics of snacks and their relationships with CMR indicators among a nationally representative sample of US adolescents. We included multiple snack characteristics and examined their associations with CMR indicators, capturing multiple dimensions of snacks and highlighting the nutritional composition of snacks. Additionally, we have controlled for confounders including meal frequency and meal nutritional quality, disentangling the effect of snack from meal and eating frequency.

The results of this study should be interpreted considering several limitations. One notable limitation is the absence of a tool specifically designed to evaluate snack nutritional quality. The nutrient profiling score used in this study was not originally designed to evaluate snack nutritional quality. Additionally, it was not intended to reflect the nutrient composition of snacks associated with cardiometabolic health. However, there is substantial overlap between the food groups and nutrients included in the HNDS and HEI-2015. This overlap suggests that the HNDS may also reflect adherence to the Dietary Guidelines for Americans. Adherence to these guidelines, as assessed by the HEI, has been associated with improvements in cardiometabolic health among adolescents^(42)^. Although the HNDS was not specifically designed to capture the nutritional composition of snacks related to optimal cardiometabolic health in adolescents, the overlap in food groups and nutrients may imply that the HNDS inadvertently includes food components related to adolescent cardiometabolic health. Future research could explore snack-specific nutritional quality indices explicitly designed to evaluate snack nutritional quality, with an emphasis on the nutrients and food groups associated with optimal cardiometabolic health in adolescents. Additionally, relying on a single day’s dietary data from a 24-h recall may not accurately capture day-to-day variations in snack consumption. The use of mean snack characteristics from multiple 24‑h recalls without methods specifically designed to estimate usual intake introduces random error. Consequently, analyses based on these unadjusted means tend to underestimate a person’s usual intake and produce attenuated associations between snack characteristics and health outcomes. Further, the use of a single 24-h recall was necessary to maximise the number of adolescents included in the study, particularly those assessed for fasting blood glucose and TAG. Furthermore, metabolic risk could not be calculated for adolescents categorised as ‘Other Race – Including Multi-Racial’ due to the lack of a metabolic risk calculation tool tailored to this population. Consequently, we excluded those in the ‘Other Race – Including Multi-Racial’ category when exploring the association between snack characteristics and CMR. While the models were adjusted for potential energy misreporting, the calculations for energy intake to estimated energy requirement (EI:EER) assumed all adolescents had a ‘low-active’ physical activity level. It is worth noting that, given the predominantly insufficient physical activity levels among US adolescents, assigning a low activity level is unlikely to significantly impact the results^(26)^.

### Conclusion

In conclusion, snack nutritional quality and snack ED (excluding beverages) were associated with overall diet quality among adolescents. Few modest associations were found between snack characteristics, cardiometabolic indicators and CMR scores in girls, while no associations were observed in boys. Further prospective research that assesses habitual dietary intake and accounts for energy misreporting is needed to draw firmer conclusions about the relationship between snack characteristics and metabolic risk factors in adolescents. Further development of snack nutritional quality indices that account for variations by sex and age and are specifically designed to predict CMR is also needed to better understand the relationship between snacking and cardiometabolic health in adolescents.

## Supporting information

Sisay et al. supplementary material 1 Sisay et al. supplementary material

Sisay et al. supplementary material 2 Sisay et al. supplementary material

Sisay et al. supplementary material 3 Sisay et al. supplementary material

## Supplementary material

For supplementary material/s referred to in this article, please visit https://doi.org/10.1017/S0007114525105746.
