# Supplementary material for: Associations of snack frequency, energy density and nutritional quality with diet quality and cardiometabolic risks in adolescents: National Health and Nutrition Examination Survey 2009–2016
Source: Br J Nutr. 2025 Nov 19;135(1):98–107. doi: 10.1017/S0007114525105746 (PMC12867603; doi:10.1017/S0007114525105746)
Supplement: Sisay et al. supplementary material 1 — Sisay et al. supplementary material [file S0007114525105746sup001.docx]

Additional File 1 Participant flowchart for inclusion in the analysis of adolescent snacking
