# Supplementary material for: Associations of snack frequency, energy density and nutritional quality with diet quality and cardiometabolic risks in adolescents: National Health and Nutrition Examination Survey 2009–2016
Source: Br J Nutr. 2025 Nov 19;135(1):98–107. doi: 10.1017/S0007114525105746 (PMC12867603; doi:10.1017/S0007114525105746)
Supplement: Sisay et al. supplementary material 2 — Sisay et al. supplementary material [file S0007114525105746sup002.docx]

Supplementary Table 2.1 Food groups (What We Eat In America Food Groups) consumed at snack among adolescents participating in NHANES 2009-2016.

| **Food Category** | **Mean(grams)** | **95% CI** |
| --- | --- | --- |
| Sugar-sweetened beverages | 171.9 | 156.0, 187.8 |
| Total milk | 55.1 | 46.9, 63.3 |
| Fruits | 31.0 | 27.3, 34.6 |
| Other desserts | 22.3 | 19.0, 25.6 |
| Mixed dishes | 20.8 | 17.8, 23.8 |
| Sweet bakery products | 19.3 | 17.1, 21.5 |
| Juice | 19.6 | 15.2, 24.1 |
| Savory snacks | 12.2 | 10.9, 13.4 |
| Diet beverages | 12.1 | 6.5, 17.8 |
| Candy | 10.1 | 8.2, 12.0 |
| Grains | 8.7 | 7.5, 9.9 |
| Vegetables | 6.2 | 4.7, 7.6 |
| Flavored milk | 6.6 | 3.7, 9.4 |
| Animal protein | 5.5 | 4.2, 6.8 |
| Milk substitutes | 4.9 | 3.2, 6.6 |
| Mixed dishes (soup) | 4.2 | 2.8, 5.6 |
| Breads | 4.3 | 3.7, 4.9 |
| Mixed dishes (pizza) | 3.6 | 2.4, 4.8 |
| Mixed dishes (Mexican) | 3.6 | 2.5, 4.7 |
| Plant protein | 2.8 | 2.2, 3.4 |
| White potatoes | 2.3 | 1.4, 3.1 |
| Cured meats | 2.1 | 1.5, 2.7 |
| Ready-to-eat cereal | 2.0 | 1.5, 2.5 |
| Crackers | 2.3 | 1.9, 2.8 |
| Cheese | 2.4 | 1.9, 2.9 |
| Snack bars | 1.4 | 1.1, 1.8 |
| Mixed dishes (sandwich) | 1.5 | 0.7, 2.3 |
| Mixed dishes (burger/frank) | 1.5 | 0.9, 2.0 |
| Quick breads | 1.2 | 0.7, 1.6 |
| Eggs | 0.7 | 0.2, 1.3 |
| Cooked cereals | 0.7 | 0.2, 1.3 |
| Yogurt | 0.8 | 0.4, 1.2 |
| Mixed dishes (grain-based) | 5.3 | 4.0, 6.5 |
| Mixed dishes (Asian) | 0.4 | 0.2, 0.6 |
| Mean: Mean intake across all snacks | | |

Supplementary Table 2.2 Contribution of snacks to food groups and contribution to overall dietary intake among adolescents participating in NHANES 2009-2016.

| **Dietary Component** | **Mean** | **95% CI** | **Contribution to overall intake (%)*** |
| --- | --- | --- | --- |
| Saturated fat (gram) | 32.2 | 30.9,33.5 | 29.4% |
| Added sugars (tsp) | 8.7 | 8.1, 9.4 | 39.5% |
| Sodium (mg) | 4.5 | 4.4,4.7 | 24.4% |
| Refined grains (Oz-eq) | 1.2 | 1.1, 1.3 | 18.0% |
| Protein foods (Oz-eq) | 0.5 | 0.4, 0.6 | 8.8% |
| Dairy (cup eq.) | 0.4 | 0.4, 0.5 | 19.6% |
| Fruits (cup eq.) | 0.3 | 0.3, 0.4 | 39.2% |
| Whole grains(oz eq) | 0.2 | 0.1, 0.2 | 21.8% |
| Vegetables (cup eq.) | 0.1 | 0.1, 0.1 | 11.6% |
| Energy (kcal) | 178.9 | 164.8, 193.0 | 22.4% |
| Mean: Mean intake across all snacks  * Contribution of snacks to the total intake of respective food group | | | |
