# Supplementary material for: Associations of snack frequency, energy density and nutritional quality with diet quality and cardiometabolic risks in adolescents: National Health and Nutrition Examination Survey 2009–2016
Source: Br J Nutr. 2025 Nov 19;135(1):98–107. doi: 10.1017/S0007114525105746 (PMC12867603; doi:10.1017/S0007114525105746)
Supplement: Sisay et al. supplementary material 3 — Sisay et al. supplementary material [file S0007114525105746sup003.docx]

Supplementary Table 3.1Characteristics of adolescents (12-19 years) with and without snack consumption in the National Health and Nutrition Examination Survey (NHANES) 2009-2016.

|  | Non-snack consumers (n=1,012) | Snack consumers (n= 3,896) | P value^1^ |
| --- | --- | --- | --- |
|  | Mean 95 % CI | Mean 95 % CI |  |
| Age (Years) | 15.5 (15.3,15.6) | 15.4 (15.2,15.5) | 0.32 |
| Race and ethnicity (%) |  |  |  |
| Mexican American | 0.1 (0.1,0.2) | 0.2 (0.1,0.2) | 0.05 |
| Other Hispanic | 0.1 (0.1,0.1) | 0.1 (0.1,0.1) |  |
| Non-Hispanic White | 0.5 (0.5,0.6) | 0.6 (0.5,0.6) |  |
| Non-Hispanic Black | 0.2 (0.1,0.2) | 0.1 (0.1,0.2) |  |
| Other Race - Including Multi-Racial | 0.1 (0.1,0.1) | 0.1 (0.1,0.1) |  |
| Ratio Family income to poverty (%) |  |  |  |
| <125% | 0.3 (0.3,0.4) | 0.3 (0.3,0.3) | 0.18 |
| ≥125% | 0.7 (0.6,0.7) | 0.7 (0.7,0.7) |  |
| Fasting blood glucose (mg/dL) | 94.3 (92.8,95.8) | 94.5 (93.5,95.5) | 0.83 |
| Triglyceride (mg/dL) | **71.7 (67.2,76.2)** | **80.8 (77.1,84.6)** | **< 0.01** |
| High density lipoprotein (mg/dL) | 51.3 (50.1,52.4) | 51.6 (51,52.3) | 0.51 |
| Low density lipoprotein (mg/dL) | 88.3 (85.3,91.4) | 87.9 (86.2,89.6) | 0.81 |
| Total cholesterol (mg/dL) | 155 (152.5,157.6) | 156.9 (155.4,158.3) | 0.21 |
| Systolic blood pressure (mmHg) | 109.4 (108.3,110.4) | 108.7 (108.2,109.2) | 0.16 |
| Diastolic blood pressure (mmHg) | 59.6 (58.4,60.7) | 59.2 (58.2,60.2) | 0.48 |
| Waist circumference (cm) | **84.5 (83.1,85.8)** | **81.6 (80.9,82.3)** | **< 0.001** |
| Metabolic syndrome risk score | 2.7 (2.7,2.8) | 2.7 (2.7,2.8) | 0.85 |
| HEI-2015 | **44.5 (43.4,45.5)** | **45.7 (45.1,46.2)** | **< 0.05** |
| BMI Z-score | **0.8 (0.7,0.9)** | **0.6 (0.5,0.6)** | **< 0.001** |
| ^1^ P-value for differences between boys and girls based on F test (continuous variable) or chi square test (categorical variables)  HEI, Healthy Eating Index | | | |

Supplementary Table 3.1: The association between snack frequency (including adolescents with no snack consumption), diet quality, cardiometabolic indicators, and Metabolic syndrome risk score among adolescents (12-19 years) in the National Health and Nutrition Examination Survey (NHANES) 2009-2016.

|  | HEI-2015^1^ | Fasting blood glucose  (mg/dL)^2^ | Triglyceride (mg/dL) ^2^ | Total cholesterol^2^ | High density lipoprotein (mg/dL) ^2^ | Low density lipoprotein  (mg/dL) ^2^ | Systolic blood pressure (mmHg) ^2^ | Diastolic blood pressure (mmHg) ^2^ | Waist circumference (cm) ^2^ | Metabolic syndrome risk score^2^ |
| --- | --- | --- | --- | --- | --- | --- | --- | --- | --- | --- |
|  | β (95% CI) | β (95% CI) | β 95% CI | β 95% CI | β 95% CI | β 95% CI | β 95% CI | β 95% CI | β 95% CI | β 95% CI |
| **Boys** |  |  |  |  |  |  |  |  |  |  |
| Snack frequency (n/day) | **0.61 * (0.12,1.09)** | -0.10  (-0.96,0.77) | 0.02  (-0.01,0.06) | 0.01  (0.00,0.02) | -0.08  (-0.61,0.44) | -0.79  (-2.93,1.36) | -0.2  (-0.7,0.29) | -0.38  (-0.82,0.07) | 0.00  (-0.01,0.00) | 0.03  (-0.02,0.08) |
| **Girls** |  |  |  |  |  |  |  |  |  |  |
| Snack frequency (n/day) | **1.04 *** (0.52,1.56)** | -0.32  (-0.82,0.17) | 0.01  (-0.03,0.04) | 0.01 (0.00,0.02) | 0.38  (-0.14,0.89) | 1.15  (-0.16,2.46) | -0.05  (-0.47,0.37) | 0.02  (-0.53,0.57) | **-0.02 *****  **(-0.03, -0.01)** | 0.01  (-0.02,0.05) |
| ^1^ These results, obtained from multiple linear regression, were further adjusted for age, race and ethnicity, survey cycle, meal frequency, waist circumference (except in model including WC as an outcome), and EI: EER.  ^2^ These results, obtained from multiple linear regression, were further adjusted for age, race and ethnicity, survey cycle, meal frequency, BMI Z score (except in model including WC as an outcome), and EI: EER.  P values: * p < 0.05, ** p < 0.01, *** p < 0.001  CI: Confidence interval  Waist circumference and triglyceride were log-transformed to improve normality. The format for interpretation of the b-coefficient estimates is therefore 100 × (coefficient), corresponding to the percentage change for a 1-unit increase in the independent variable (while holding all other variables constant). | | | | | | | | | | |
